# Editorial: The role of monocytes/macrophages in autoimmunity and autoinflammation

**DOI:** 10.3389/fimmu.2022.1093430

**Published:** 2022-11-22

**Authors:** Atsushi Kawakami, Naoki Iwamoto, Keishi Fujio

**Affiliations:** ^1^ Department of Immunology and Rheumatology, Division of Advanced Preventive Medical Sciences, Nagasaki University Graduate School of Biomedical Sciences, Nagasaki, Japan; ^2^ Department of Allergy and Rheumatology, Graduate School of Medicine, The University of Tokyo, Tokyo, Japan

**Keywords:** monocyte - macrophage, autoinflammation disease, autoimmune disease, rheumatoid arthritis, systemic lupus - erythematosus, vasculitis

Monocytes and macrophages are widely distributed innate immune cells that play indispensable roles in a variety of physiologic and pathologic processes, including regulation of the initiation, development and resolution of inflammatory disorders. They also actively participate in the development of autoimmune and autoinflammatory diseases. Their active mechanism is essentially to secrete a wide range of cytokine and chemokines, leading to the recruitment of additional specific immune cells.

Abnormal regulation of monocytes and macrophages has been reported in several autoimmune diseases. An example of this is the infiltrating CD16^+^ are associated with a reduction in peripheral CD14^+^CD16^+^ monocytes in systemic lupus erythematosus (SLE) patients (1). Macrophages from SLE patients overexpress intercellular adhesion molecule (ICAM-1), and this overexpression is mitigated by corticosteroid treatment (2). Unusual expression of inflammatory and regulatory molecules associated with monocytes have also been found in autoinflammatory diseases, such as microRNA-204-3p in familial Mediterranean fever (3).

Furthermore, macrophages can be phenotypically polarized by the surrounding microenvironmental stimuli and signals. Classically activated macrophages (M1) can produce toxic effector molecules such as reactive oxygen species and nitric monoxide, as well as inflammatory cytokines such as interleukin (IL)-1β, tumor necrosis factor (TNF) and IL-6. Alternatively activated macrophages (M2) drive immune regulation, tissue remodeling. It has been suggested that such macrophage and monocyte subset polarization contributes to autoimmune and autoinflammatory diseases, most notably, Rheumatoid Arthritis (RA) and Crohn’s Disease (4, 5).

Interestingly, a recent investigation of immune profiling analysis linked to Genome-Wide Association Studies (GWAS) has suggested the importance of monocyte-macrophage lineage cells for the development of autoimmune and autoinflammatory conditions. As mentioned above, there is growing evidence that monocytes and macrophages affect the progression and development of autoimmune and autoinflammatory diseases. Until now, the pathogeneses of these diseases have not been fully elucidated despite the enthusiasm of researchers for this topic; moreover, few treatment options have been available for these diseases. Investigating and understanding the role of monocytes/macrophages in these diseases is likely to improve this situation.

The goal of this Research Topic was to gather Original Research articles and Reviews that have improved our understanding of monocytes and macrophages in autoimmune and autoinflammatory disease.

Eighteen articles were contributed to this edition (8 original research and 10 reviews). The original research focused on monocytes/macrophages in various autoimmune disease such as SLE, RA and vasculitis from a “bench-to-clinic” standpoint.

Murakami et al. and Nomura et al. reported on the roles of monocytes in lupus model mice. Murakami et al. revealed that inhibition of toll-like receptor 7 (TLR7) suppressed the progression of lupus nephritis in lupus-prone NZBWF1 mice. In their study, the anti-TLR7 antibody reduced IgG deposition in the glomeruli, and this reduction was caused by a decrease of patrolling monocytes. NZBWF1 mice had an abundance of Ly6C^low^ patrolling monocytes expressing a high level of TLR7 and upregulated expressions of IL-10, CD115, CD31 and TNF superfamily member (TNFSF)15, which has been associated with nephritis. Further, administration of an anti-TLR7 antibody abolished this abundance of lupus-associated patrolling monocytes. Thus, targeting of TLR7 was revealed as a promising therapeutic option targeting monocytes in lupus nephritis. Interestingly, association of TLR-7 and monocytes in a lupus model was also reported by Nomura et al. Imiquimod, a TLR-7 agonist, induces a lupus like-phenomenon in C57BL/6 and NZB/NZW mice by activating TLR-7 signaling. The authors studied the involvements of different monocytes in these model mice according to site and temporal disease progression. Ly6C^hi^ monocytes were increased in the lymph nodes and upregulated interferon (IFN)-α genes, whereas Ly6C^lo^ monocytes were increased in the late phase, infiltrating tissues and becoming inflammatory cells in the kidneys. In human SLE patients, Zhu et al. reported that the level of ribonuclease A family member 2 (RNASE2), which is known to have antiviral activity and immunomodulatory function, was associated with the proportion of autoreactive B-cells, and expansions of these cells were related with monocyte-derived IL-10 levels. mRNA expression of RNASE2 was elevated in peripheral blood mononuclear cells from SLE patients and was particularly associated with autoreactive B-cell subsets. Moreover, silencing of RNASE2 reduced monocyte-derived IL-10.

Recent years have seen an explosion of research focusing on macrophage polarization in autoimmune disease. We gathered five articles addressing macrophage polarization. Paoletti et al. investigated differences in macrophage polarization capabilities according to treatment. They reported that polarization into M2 macrophage from monocytes of RA patients *in vitro* was decreased compared with that of healthy donors. Interestingly, this *in vitro* defect in monocyte polarization into M2 macrophages was restored in monocytes from RA patients treated with adalimumab, but not in those treated with etanercept. Cutolo et al. reviewed this compelling issue of macrophage polarization in relation to RA synovitis. In the review, the authors suggested that in RA, M1/Th1 activation occurs in an inflammatory environment dominated by TLR and IFN signaling, promoting the abundant production of pro-inflammatory cytokines and matrix metalloproteinases, resulting in osteoclastogenesis and the progression of joint destruction; on the other hand, the activation of M2/Th2 promotes the release of growth factors and cytokines involved in the anti-inflammatory process leading to clinical remission of RA. They reasoned that since the synovial tissue of RA under remission is characterized by a higher presence of M2 macrophages, the regulation of M1/M2 imbalances in favor of anti-inflammatory M2 macrophages might represent a clear therapeutic goal in the management of RA. Bibliometric analysis by Xu et al. quantified the existing research on the roles of macrophages in RA from 2000 to 2021. During that period a total of 7253 original articles related to macrophages in RA were published. In the publications on this subject, “bone loss” and “polarization” were the most frequently used keywords. These authors certainly confirmed that the study of macrophage polarization in RA has become a keen research focus in recent years.

The association of macrophage polarization with inflammatory bowel disease (IBD) was reported by Tian et al. Astragaloside IV (AS- IV), a natural saponin extracted from the traditional Chinese medicine herb *Ligusticum chuanxiong*, attenuated the clinical activity of dextran sulfate sodium (DSS)-induced colitis, which mimics human IBD. That is, AS-IV administration modulated the phenotype change of macrophages, inhibiting pro-inflammatory macrophages and promoting pro-resolution macrophages to ameliorate the progression of DDS-induced colitis *via* the regulation of the STAT signaling pathway. Hirahara et al. reviewed the role of monocytes/macrophages in Behҫet’s disease (BD), also with a focus on macrophage polarization. They summarized the role of monocytes/macrophages in the pathogenesis of BD, including the issue of genetic factors, and discussed the abnormal macrophage polarization in the context of IL10, also the influence on monocytes of current BD treatments.

He et al. analyzed monocyte subsets in patients with Sjögren’s syndrome (SS) and controls. Single-cell RNA sequencing using monocytes from SS patients identified a new monocyte subset characterized by higher expression of VNN2 and S100A2, and this subset was increased in SS patients. Further, all monocyte subsets from SS patients had increased expression of TNFS10, and the IFN-related and neutrophil activation-associated pathways were also upregulated in SS-derived monocytes.

Regarding vasculitis, we present one original study and one review. Tang et al. reported how extravasation of CD16^+^monocytes to the kidneys was involved in renal damage in myeloperoxidase ANCA-associated vasculitis (MPO-AAV). An increase of CD16^+^monocytes in the glomeruli of MPO-AAV patients with renal damage was detected, and this increase was supposed to be caused by the MPO-ANCA promotion of an increase in the C-X3-C motif chemokine ligand 1 (CX3CL1) on glomerular endothelial cells, leading to recruitment of CD16^+^monocytes. The pathogenic role of monocytes/macrophages in large vessel vasculitis (LVV) was reviewed by Watanabe and Hashimoto They focused on the subpopulation of circulating monocytes and tissue macrophages in LVV and discussed the potential blockades of chemokines or chemokine–receptor interactions that attract circulation monocytes and T cells as a therapeutic option. However, they noted that monocytes might have already been recruited to the vascular tissues and differentiated into macrophages by time LVV has developed.

Because circulating monocytes might migrate to a specific location in the bones and fuse with each other to become mature multinucleated osteoclasts, monocytes are important sources of osteoclasts. We have two review articles addressing this issue. Hasegawa and Ishii reviewed the heterogeneity of osteoclasts involved in inflammatory arthritis. Their single-cell RNA sequence analysis of the osteoclast precursor-containing macrophages in the joints succeeded in specifying the populations that differentiate into mature osteoclasts at the arthritic inflammation site (pannus-bone interface), called “arthritis-associated osteoclastogenic macrophages (AtoMs)”. They also discussed the clinical implications of AtoMs, such as factors which most effectively differentiate AtoMs into osteoclasts, specifically receptor activator of nuclear factor-kappa B ligand (RANKL) and TNF. A systematic review by Zuo and Deng summarized the role in osteoclastogenesis of Fc gamma receptors (FcγRs), which are expressed on the surfaces of monocytes and macrophages. The activation of FcγRs is required for RANKL-induced osteoclastogenesis; thus, FcγRs can regulate inflammatory arthritis. The FcγRs may have dual roles in osteoclastogenesis, i.e., both inhibiting and activating, depending on the extent of FcγR occupancy by IgG and RANKL. For example, specific IgG molecules of Fc fragments with a high affinity to FcγRs designed to occupy FcγRI may inhibit osteoclastogenesis.

Kamata and Tada reviewed the roles of dendritic cells and macrophages in the pathogenesis of psoriasis. They focused on plasmacytoid dendritic cells (pDCs) and M1 macrophages in the early phase of psoriasis. pDCs produce IFN-α, causing the maturation of resident dermal DCs and the differentiation of monocytes into inflammatory DCs, which produce the key cytokines of psoriasis. In early-phase psoriasis, M1 macrophages contribute to development of psoriasis by producing TNF-α

Tsuboi et al. reviewed the activation mechanism of monocytes/macrophages into adult-onset Still disease (AOSD). In this review, they discussed monocyte/macrophage activation by several factors such as the pathogen-associated molecular pattern (PAMPs), damage-associated molecular patterns (DAMPs), and neutrophil extracellular traps (NETs)-DNA. These stimulation plays an important role in the pathogenesis of AOSD by causing activation of the nucleotide-binding oligomerization domain and the pyrin domain (NLRP) 3 inflammasomes, which trigger caspase-1 activation, resulting in the conversion of pro-IL-1β and pro-IL-18 into their mature forms. They also identified placenta-specific 8 (PLAC8) as a molecule whose expression is elevated in the monocytes of active AOSD.

A comprehensive overview of current advances in the use of induced pluripotent stem cell (iPSC)-derived monocytes/macrophages for research into autoinflammatory diseases was provided by Tanaka et al. They discussed the possibility of immortalized PSC-derived cell lines produced by introducing *MYC, BML2* and *MDM2* into iPSC-derived floating monocytic cells, and their potential use for research on disease pathogenesis.

The role of monocytes/macrophages in the disease development or progression of autoimmune and autoinflammatory disease can be described as “trained immunity”; for example, some vaccines and microorganisms induce epigenetic changes in monocytes/macrophages, modifying their functional response. In this regard, Funes et al. reviewed the relationship of trained immunity to autoimmune and inflammatory disorders.

Lastly, Nagafuchi et al. summarized their results from transcriptome analysis of various autoimmune diseases. Transcriptome analysis enables researchers to observe the dynamics of gene expression in different cell types. They focused on their recent studies using immuNexUT, a database containing immune cell gene expression data from various immune-mediated diseases and many types of immune cells, in addition to healthy controls. In particular, they discussed how single-cell RNA-seq analysis has provided atlases of infiltrating immune cells in various autoimmune disease lesions, and also how expression quantitative trait locus (eQTL) analysis can help identify candidate causal genes and immune cells.

In conclusion, we are pleased that this issue focusing on the Research Topic “*the role of monocytes/macrophages in autoimmunity and autoinflammation*” has attracted a variety of novel investigations and discussions of current research on monocytes/macrophages that should provide interested readers a wealth of insights on the links between monocyte-macrophage lineage cells and autoimmune/autoinflammatory diseases.

## Author contributions

AK, NI and KF have completed this editorial articles. All authors made substantial, direct and intellectual contribution to the work. All authors have given their final approval of the manuscript to be published as presented.

## Conflict of interest

The authors declare that the research was conducted in the absence of any commercial or financial relationships that could be construed as a potential conflict of interest.

## Publisher’s note

All claims expressed in this article are solely those of the authors and do not necessarily represent those of their affiliated organizations, or those of the publisher, the editors and the reviewers. Any product that may be evaluated in this article, or claim that may be made by its manufacturer, is not guaranteed or endorsed by the publisher.
